# Immunocytokines in cancer: a safety perspective

**DOI:** 10.3389/fimmu.2026.1894312

**Published:** 2026-09-29

**Authors:** Kushal Prajapati, Catherine Betts, Richard Stebbings

**Affiliations:** 1 Safety Sciences, Clinical Pharmacology and Safety Sciences, R&D, AstraZeneca, Gaithersburg, MD, United States; 2 Safety Sciences, Clinical Pharmacology and Safety Sciences, R&D, AstraZeneca, Cambridge, United Kingdom

**Keywords:** cancer, cytokines, immunocytokines, immunogenicity, immunotherapy, immunotoxicology, safety, translational safety

## Abstract

Immunocytokines (ICs) are engineered biologics comprising a fusion of cytokines to antibodies, designed to deliver targeted cytokines to diseased tissues and mitigate the severe safety challenges associated with systemic cytokine therapy. A rapidly growing number of clinical trials have evaluated ICs, largely based on IL-2, IL-12, and TNF-α, across multiple cancer indications. Although these therapeutics often show improved safety profiles compared to systemic cytokine therapy, clinically relevant toxicities can still occur, including vascular leak syndrome (VLS), hypotension, cytokine release syndrome (CRS), cytopenias, hepatic injury, and local antigen-directed inflammatory toxicities. Although many of these adverse events are manageable, further attenuation is essential to achieve optimal efficacious dosing and to enable combination regimens. Mechanistically, IC toxicities arise through the contribution of both the antibody-mediated target binding and the cytokine moiety. These dual contributors therefore necessitate modality-specific safety considerations. In this focused narrative review, we discuss safety risks associated with clinically advanced IL-2, IL-12 and TNF-α-based ICs, outline potential underlying mechanisms and highlight cytokine- and format-specific factors. We further discuss selected non-clinical mitigation strategies, immunogenicity and translational safety considerations, and outline future directions for developing safer next-generation ICs.

## 1 Introduction

Immunocytokines (ICs) are cytokines conjugated to an antibody to enable targeted delivery, with the aim of mitigating toxicities associated with systemic administration and thereby maximizing the therapeutic index. The genesis of ICs can be traced back to investigational use of cytokines for cancer therapy around 1990, when it became evident that systemic IL-2 administration was associated with incidences of potentially lethal vascular and cardiorespiratory toxicities in patients (1, 2). This led multiple groups to begin fusing IL-2 with antibodies targeting tumor-associated antigens (TAAs) such as epithelial cell adhesion molecule (EpCAM), disialoganglioside (GD2), and extracellular matrix/stromal components such as fibronectin to enable targeted cytokine delivery (3–7). Since then, this emerging modality has significantly expanded to include diverse cytokines and TAAs with a rapidly growing number of clinical trials underway for both solid and hematologic cancers (8). Clinically, ICs have been evaluated in multiple cancer indications, often as second or third line therapies via routes such as intratumoral (9), intravenous (5), isolated limb perfusion (ILP) (10) and subcutaneous (11). ICs have been administered either as monotherapy (9, 12) or in combination with chemotherapy (13, 14), immunotherapy (15, 16) or radiotherapy (8). This approach has shown promise in both preclinical and clinical settings.

Owing to their tumor-targeting antibody component, ICs have generally shown an improved safety profile compared to historical experience with systemic cytokine administration (8). ICs can also improve the pharmacokinetic profile of free cytokines, potentially allowing for lower or less frequent, more tolerable doses compared to systemic cytokines (17–19). For instance, high-dose IL-2 therapy has been historically associated with severe (grade ≥3) hypotension arising from vascular leak syndrome (VLS) (20–23), whereas targeted IL-2 ICs have generally been associated with mild to moderate (grade 1–2) hypotension across various trials (5, 6, 13, 24–27). Similarly, systemic TNF-α therapy is associated with severe hypotension, shock, and multi-organ failure in cancer patients (28), whereas targeted TNF-α IC administration via ILP showed a dramatically improved therapeutic index with no systemic shock (10, 29). Likewise, administration of targeted IL-12 IC has demonstrated better tolerability and enabled substantially higher dose levels than had historically been feasible with systemic IL-12 administration (16, 30). Despite the promise of ICs for alleviating safety concerns associated with traditional systemic cytokine therapies, they still carry the same core risks and in fact can potentially present new safety challenges attributed to their design. Available clinical data from ICs show hematological changes including vascular events (vascular leak, hypotension), cytopenias, cytokine release syndrome (CRS)-related symptoms (flu/fever), infusion-related reactions, metabolic imbalance (hypophosphatemia, hyperglycemia) and local inflammatory effects (skin rash, injection-site reactions) among the major adverse events (AEs) reported in patients (8). Many of these toxicities are reversible or manageable in the clinic with premedication or supportive care. However, further attenuation of these dose-limiting toxicities (DLT) is crucial to maximize IC therapeutic efficacy and allow combination with other immunomodulatory or chemotherapy agents. Hence, alleviating toxicities and augmenting the maximum tolerated dose (MTD) are imperative to realize the true potential of ICs in treating cancer.

In this safety-focused review, we discuss key safety risks associated with IL-2, IL-12, and TNF-α ICs, provide mechanistic insights wherever supported by evidence, and assess cytokine-, route- and modality-specific factors that may contribute to these toxicities. We also highlight selected mitigation strategies drawn from emerging clinical and preclinical literature, discuss immunogenicity and translational safety aspects, and offer future perspectives on the development of safer ICs for cancer therapy.

## 2 Safety risks associated with ICs

Many of the characteristic toxicities seen with ICs in the clinic can be attributed to the cytokine moiety, mirroring toxicities seen with lower doses of systemic cytokines. However, the magnitude and pattern of risk are also shaped by the route of administration and by antibody-associated factors, including target expression in normal tissues, pharmacokinetics (PK), target-mediated local retention, and Fc-mediated effector mechanisms. Here, we focus discussion on the primary clinical toxicities observed with IL-2, IL-12 and TNF-α ICs, as these cytokines are among the most clinically advanced and provide the clearest safety signals. A cross-trial summary of selected clinical safety findings, including grade ≥3 treatment-related adverse events, dose-limiting toxicities, reported MTD or recommended phase 2 dose (RP2D) values, and current development status is provided in **Table 1**. Some agents discussed below are no longer in active clinical development, but their reported safety profiles remain informative for IC safety assessment.

**Table 1:** Cross-trial summary of selected immunocytokines clinical studies in cancer, including grade ≥3 treatment-related adverse events, dose-limiting toxicities, and MTD/RP2D.

Immunocytokine	Cytokine	Target	Disease/setting	Reference	Trial number	Dosing route and frequency	Dose level(s) evaluated	N treated	Treatment related grade ≥3 AEs (n/N, %)	Dose-limiting toxicities (n/N, %)	MTD/RP2D	Development status
hu14.18-IL2 (EMD 273063)	IL-2	GD2	Metastatic melanoma	(5)	NR	4h IV infusion on day 1–3 every 4–5 weeks	0.8, 1.6, 3.2, 4.8, 6.0, 7.5 mg/m²/day	33	Hypophosphatemia (11/33, 33%) Hyperglycemia (3/33, 9%) Hypotension (2/33, 6%) Hypoxia (3/33, 9%) Elevated hepatic transaminases (2/33, 6%) Thrombocytopenia (1/33, 3%) Hyperbilirubinemia (1/33, 3%)	DLTs at 7.5 mg/m²/d: Hypoxia (1/6, 17%) Hypotension (1/6, 17%) Elevated AST/ALT (1/6, 17%) Hyperglycemia (1/6, 17%) Total (4/6, 67%)	MTD: 7.5 mg/m²/day	Discontinued
hu14.18-IL2 (EMD 273063)	IL-2	GD2	Metastatic melanoma	(94)	EMR 62207-005	4h IV infusion on day 1–3 every 4 weeks	4 mg/m²/day	9	Total grade ≥3 AEs (6/9, 67%) Treatment related grade 3 AEs: Rigors (3/9, 33%), Pyrexia (2/9, 22%), ALT increase (1/9, 11%), Hyponatremia (1/9, 11%) Hypoxia (1/9, 11%) Hypokalemia (1/9, 11%) Rash (1/9, 11%)	No formal DLT criteria defined	MTD: 7.5 mg/m²/day (previously established)	Discontinued
hu14.18-IL2 (EMD 273063)	IL-2	GD2	Metastatic melanoma	(48)	NCT00109863	4h IV infusion on day 1–3 every 4 weeks	6 mg/m²/day	14	Lymphopenia (13/14, 92%) Hypophosphatemia (5/14, 35%) Thrombocytopenia (3/14, 21%) Hyperglycemia (2/14, 14%) Hyponatremia (1/14, 7%) Hypokalemia grade 3 (1/14, 7%) Hypermagnesemia (1/14, 7%)	Hypotension (1/14, 7%)	MTD: 7.5 mg/m²/day (previously established)	Discontinued
hu14.18-IL2 (EMD 273063)	IL-2	GD2	Stage III or stage IV melanoma	(24)	NCT00590824	4h IV infusion on day 1–3 every 4 weeks	6 mg/m²/day	20	AST elevation (2/20, 10%) Elevated bilirubin (1/20, 5%) Pain (1/20, 5%)	Hypotension (3/20, 15%) Syncopal episode (1/20, 5%) Elevated AST (2/20, 10%) Elevated bilirubin (1/20, 5%) Elevated creatinine (1/20, 5%) Pain (1/20, 5%)	MTD: 7.5 mg/m²/day (previously established)	Discontinued
NHS-IL2 (EMD 521873/selectikine)	IL-2	Necrotic DNA/histone complexes	Advanced solid tumors	(55)	EudraCT No. 2006-002083-26	1h IV infusion on day 1–3 every 3 weeks	0.075–0.9 mg/kg (a subgroup received low-dose CTX 300 mg/m² on day 1 of each cycle)	48 (39 alone + 9 with CTX)	Rash (5/48, 10%) Lymphopenia (11/48, 23%) Treatment discontinued (14/48, 29%)	Rash (7/48, 14%)	MTD: 0.6 mg/kg; RP2D: 0.45–0.6 mg/kg	Discontinued
NHS-IL2 (EMD 521873/selectikine)	IL-2	Necrotic DNA/histone complexes	Metastatic NSCLC	(56)	NCT00879866	1h IV infusion on day 1–3 every 3 weeks	3 + 3 dose escalation: 0.15, 0.30, 0.45 mg/kg; preceded by RT 5×4 Gy	13	Dyspnea (2/13, 15%) Other mostly grade 1–2 toxicities: Fatigue (7/13, 53%) Rash (6/13, 46%) Anorexia (5/13, 38%) Hyperthyroidism (3/13, 23%)	Pericarditis attributed to progressing disease at 0.45 mg/kg (1/7, 14%); no DLTs at 0.15 and 0.30 mg/kg	MTD not reached; highest dose tested 0.45 mg/kg well tolerated	Discontinued
L19-IL2 (darleukin)	IL-2	EDB-fibronectin	Metastatic melanoma	(49)	NCT01055522	1h IV infusion on day 1,3,5 every 3 weeks + 1 g/m^2^ DTIC on day 1	Dose escalation 10, 15, 22.5 Mio IU, then RP2D 22.5 Mio IU	32	Leukopenia/neutropenia/anemia (7/32, 21%) Hypotension (3/32, 9%) Embolism (1/32, 3%) Pyrexia (1/32, 3%) Fatigue (1/32, 3%) Pain (1/32, 3%) Nausea (1/32, 3%) Drug intolerance (1/32, 3%) Thrombosis (1/32, 3%) Arrhythmia (1/32, 3%) Tachycardia (1/32, 3%) Metabolic and laboratory disorders (2/32, 6%)	No DLTs observed	MTD and RP2D confirmed same (22.5 Mio IU) as monotherapy	Active
L19-IL2 (darleukin)	IL-2	EDB-fibronectin	Metastatic melanoma	(13)	NCT01055522, EudraCT No. 2007-005737-11	Arm 1: 1 h IV infusion days 1,3,5 every 3 weeks for 4 cycles, then weekly + DTIC 1 g/m² on day 1 of each cycle; Arm 2: 1 h IV infusion weekly + DTIC on day 1 of each cycle; Arm 3: DTIC alone on day 1 every 3 weeks	22.5 Mio IU	67 (22 Arm 1 + 22 Arm 2 + 23 Arm 3)	Arms 1 + 2 combined: Neutropenia (5/44, 11%) Thrombocytopenia (4/44, 9%) ALT increase (2/44, 5%) Arthralgia (3/44, 7%) GGT increase (2/44, 5%)	NR	RP2D: 22.5 Mio IU (established in prior studies)	Active
L19-IL2 (darleukin)	IL-2	EDB-fibronectin	Oligometastatic tumors	(50)	NCT02086721	SBRT then 3-h IV infusion of L19-IL2 within 1 week on day 1,3,5 for a 3-week cycle	3 + 3 dose escalation: 15 and 22.5 Mio IU	6 (3 for each dose)	GGT elevation at 22.5 Mio IU (2/3, 66%) ALT elevation at 22.5 Mio IU (1/3, 33%) Syncope at 22.5 Mio IU (1/3, 33%)	Liver toxicity (2/3, 66%) Vasovagal syncope (1/3, 33%) at 22.5 Mio IU; no DLT at 15 Mio IU	RP2D: 15 Mio IU (in combination with SBRT)	Active
L19-IL2 (darleukin)	IL-2	EDB-fibronectin	Advanced metastatic melanoma	(51)	NCT01253096	Intralesional injection weekly for 4 weeks	10 Mio IU/injection	25	Injection site reaction (unspecified number of patients) Injection pain (1/25, 4%) Transient fatigue (1/25, 4%)	No formal DLT criteria defined	No formal MTD/RP2D defined in intralesional setting	Active
L19-IL2 (darleukin)	IL-2	EDB-fibronectin	Advanced renal cell carcinoma	(7)	NCT01058538	1-h IV infusion on day 1, 3, 5 every 3 weeks	Dose escalation phase I: 5, 10, 15, 22.5, 30 Mio IU; expansion phase II at 22.5 Mio IU	33 (21 phase I + 12 phase II)	Expansion phase: Hypertension (2/12, 17%) Hypoxia (2/12, 17%) Vascular (1/12, 8%) Dyspnea (1/12, 8%) Pulmonary (2/12, 17%) Cardiac (2/12, 17%) Gastrointestinal (1/12, 8%) Skin (1/12, 8%) Metabolic/laboratory (1/12, 8%) Lymphatics (1/12, 8%) Blood/bone marrow toxicity (5/12, 42%) Escalation phase: Neurology (1/21, 5%) at 30 Mio IU	No formal DLTs at doses ≤22.5 Mio IU; study closed at 30 Mio IU DLTs at 22.5 Mio IU: Capillary leak (1/21, 4%), Renal failure (1/21, 4%) DLTs at 30 Mio IU: Syncope (1/21, 4%), Elevation in serum creatinine (1/21, 4%)	MTD: >22.5 Mio IU (not formally exceeded with DLT criteria); RD: 22.5 Mio IU	Active
F16-IL2	IL-2	Tenascin-C domain A1	Advanced solid tumor; Expansion in metastatic breast cancer	(69)	NCT01131364	1-h IV infusion on days 1, 8, 15 every 4 weeks	5–25 Mio IU + DOX (0–25 mg/m²) RP2D phase II: F16-IL2–25 Mio IU + DOX 25 mg/m²	29 (19 phase I + 10 phase II)	Blood/lymphatic disorders (9/29, 31%) General disorders and administration site conditions (4/29, 13%) Metabolism/nutrition disorders (2/29, 6%) Gastrointestinal disorders (1/29, 3%) Febrile neutropenia (1/29, 3%)	No DLTs observed	RP2D: 25 Mio IU + DOX (25 mg/m²)	Active
huKS-IL2 (EMD 273066)	IL-2	EpCAM	Advanced solid tumors	(27)	NCT00132522	4-h IV infusion days 2–4 every 3 weeks for up to 6 cycles	0.5, 1.0, 2.0, 3.0, 4.0 mg/m²/day, preceded by 300 mg/m² CTX on day 1	27	Grade ≥3 AEs (11/27, 41%) Lymphopenia (1/27, 4%) Hypophosphatemia (2/27, 7%) Anemia (1/27, 4%) Thrombocytopenia (1/27, 4%) Leukopenia (1/27, 4%) Phosphorous equivalents increase (1/27, 4%) GGT increase (1/27, 4%) Ascites (1/27, 4%) Nausea (1/27, 4%) Vomiting (1/27, 4%) Dyspnea (1/27, 4%) Bronchospasm (1/27, 4%) Hypoxia (1/27, 4%)	Total DLTs (4/27, 15%) Hypoxia (1/27, 4%) Dyspnea (1/27, 4%) Bronchospasm (1/27, 4%) GGT increase (1/27, 4%) Thrombocytopenia (1/27, 4%) Neutropenia (1/27, 4%) Anemia (1/27, 4%)	MTD: 3.0 mg/m²/day	Discontinued
huKS-IL2 (EMD 273066)	IL-2	EpCAM	Small cell lung cancer	(26)	EudraCT No. 2006-004566-13	4-h IV infusion on days 2–4 every 3 weeks until disease progression	1.5 mg/m², preceded by CTX 300 mg/m² on Day 1 of every cycle	108 randomized (64 treated with huKS-IL2 and CTX + 44 with best supportive care)	Total grade ≥3 AEs (31/64, 48%); nature of grade ≥3 AEs not specified but most common AEs of any grade were: Lymphopenia (27/64, 42%) Pyrexia (24/64, 37%) Skin disorders (22/64, 34%)	NR (phase II maintenance trial)	NR (phase II maintenance trial)	Discontinued
FAP-IL2v (Simlukafusp alfa)	IL-2 variant	FAP	Advanced/metastatic solid tumors	(25)	NCT02627274	IV infusion once every week	Flat dosing (5, 10, 20, 25 mg) Intraparticipant up titration (15/20, 20/25, 20/20/35, 20/35/35 mg)	61	Total grade ≥3 AEs (54/61, 89%) Most common grade ≥3 AEs: IRR (13/61, 21%) Anemia (10/61, 16%) AST increase (8/61, 13%) Pyrexia (7/61, 12%), Hypophosphatemia (7/61, 12%)	Total DLTs (5/61, 8%) Drug-induced liver injury (2/61, 3%) AST increase (2/61, 3%) Fatigue (1/61, 1%) Asthenia (1/61, 1%) Pneumonia (1/61, 1%)	MTD and recommended expansion dose: 15/20 mg up titration regimen	Discontinued
FAP-IL2v (Simlukafusp alfa)	IL-2 variant	FAP	Advanced solid tumors	(67)	jRCT2080224653	IV once every week for first 4 week cycle, then once every 2 weeks from cycle 2	Cohort 1: 10 mg alone Cohort 2: 10 mg + atezolizumab 840 mg every 2 weeks Cohort 3: Up titration to 15/20 mg	11	Total grade ≥3 AEs of any class (11/11, 100%) Infusion-related reaction (1/11, 9%) Lymphopenia (11/11, 100%) AST increase (4/11, 36%) ALT increase (1/11, 9%) Leukopenia (2/11, 18%) GI disorders (2/11, 18%) Blood/lymphatic disorders (4/11, 36%) Vascular disorders (1/11, 9%) Lymphocyte increase (1/11, 9%)	Hypotension (1/11, 9%) at 15/20 mg up titration	MTD/RP2D not formally established (study terminated early due to portfolio deprioritization)	Discontinued
CEA-IL2v (Cergutuzumab amunaleukin)	IL-2 variant	CEA	Advanced/metastatic CEA+ solid tumors (primarily colorectal; also RCC and melanoma cohort)	(187)	NCT02004106 (BP28920)	2-h IV infusion; Part I: every 2 weeks; Part II: every 1 or 2 weeks	Part I: 0.1–6 mg Part II: 10–40 mg	60 (5 part I + 55 part II)	Total (36/60, 60%); Treatment related grade ≥3 AEs not specified, but most frequent grade ≥3 AEs reported were: IRR (11/60, 18%) GGT increase (8/60, 13%) Hypophosphatemia (7/60, 12%) Fatigue (6/60, 10%)	Hypophosphatemia (1/60, 2%) Thrombocytopenia (2/60, 3%) Fatigue (1/60, 2%) Oliguria (1/60, 2%) LFT elevation (1/60, 2%) Dyspnea (1/60, 2%) Hypotension (1/60, 2%)	MTD: 30 mg every 2 weeks	Discontinued
L19-TNFα (Onfekafusp alfa)	TNF-α	EDB-fibronectin	Locally advanced extremity melanoma	(10)	NCT01213732	Isolated limb perfusion	325 and 650 µg (corresponding to a TNF activity of 125 and 250 µg) + melphalan (10 mg/L limb volume)	17	Leukopenia (6/17, 35%) Neutropenia (4/17, 24%) Lymphopenia (1/17, 6%) Hemoglobin decrease (2/17, 12%)	No formal DLT criteria defined	Preset doses (325 μg and 650 μg); 650 μg selected for further development	Active
NHS-IL12 (M9241)	IL-12	Necrotic DNA/histone complexes	Metastatic solid tumors	(16, 203)	NCT01417546	Subcutaneous injection once (single-dose cohort) or every 4 weeks (multiple dose cohort)	Single-dose cohort: 0.1, 0.5, 1,2,4,8,12 µg/kg Multiple dose cohort: 2,4,8,12,16.8,21.8 µg/kg	59 (22 single-dose + 37 multiple dose)	Lymphopenia (6/59, 10%) Neutropenia (4/59, 6%) Leukopenia (2/59, 3%) ALT increase (3/59, 5%) Alkaline phosphatase increase (4/59, 6%) AST increase (1/59, 1%) Lipase increase (1/59, 1%) Hypokalemia (1/59, 1%) Hyperhidrosis (1/59, 1%) At least one grade ≥3 TRAE seen (12/59, 20%)	AST/ALT elevation (3/59, 5%)	MTD/RP2D: 16.8 µg/kg	Active
CD38-IFNα (Modakafusp alfa)	IFN-α	CD38	Phase Ib: advanced/metastatic solid tumors; Phase II: unresectable/metastatic cutaneous melanoma (anti-PD-1 resistant)	(175)	NCT04157517	IV every 3 weeks	Phase Ib: dose escalation 0.1-1.5 mg/kg (6 cohorts); Phase II: 1 mg/kg + pembrolizumab 400 mg every 6 weeks	45 total (21 phase Ib + 24 phase II)	Phase Ib: Neutropenia (3/21, 14%), Thrombocytopenia (1/21, 5%) Phase II: Infusion reactions (2/24, 8%) Neutropenia (5/24, 21%) Nausea (2/24, 8%)	Phase Ib: Thrombocytopenia (1/21, 5%) Confusional state (1/21, 5%) at 1 mg/kg Phase II: No DLTs observed	RP2D: 1 mg/kg	Active

• Cross-trial comparisons are descriptive only and should be interpreted cautiously because studies differed in tumor type, population, design, DLT definition, schedule, route, and AE reporting practices. The table emphasizes variables most consistently reported across studies: grade ≥3 TRAEs, DLTs, and MTD/RP2D. Event frequencies are shown as (n/N, %) where available; denominators may vary across endpoints.

• Several agents listed here are no longer in active development or were discontinued for strategic or efficacy-related reasons. Their inclusion is intended to capture reported clinical safety observations rather than to imply ongoing development status. Development status reflects publicly available information assessed as of August 2026 and may change over time.

AE, adverse event; ALT, alanine aminotransferase; AST, aspartate aminotransferase; CD38, cluster of differentiation 38; CEA, carcinoembryonic antigen; CTX, cyclophosphamide; DLT, dose-limiting toxicity; DOX, doxorubicin; DTIC, dacarbazine; EDB, extra-domain B; EpCAM, epithelial cell adhesion molecule; EudraCT, European Union Drug Regulating Authorities Clinical Trials; FAP, fibroblast activation protein; GD2, disialoganglioside GD2; GGT, gamma-glutamyl transferase; GI, gastrointestinal; IFN-α, interferon alpha; ILP, isolated limb perfusion; IRR, infusion-related reaction; IV, intravenous; jRCT, Japan Registry of Clinical Trials; LFT, liver function test; Mio IU, millions of international units; MTD, maximum tolerated dose; NR, not reported; NSCLC, non-small cell lung cancer; RCC, renal cell carcinoma; RD, recommended dose; RP2D, recommended phase 2 dose; RT, radiotherapy; SBRT, stereotactic body radiotherapy; TRAE, treatment-related adverse event.

### 2.1 Vascular leakage/hypotension

Cytokine-driven endothelial injury is the primary mechanism of vascular toxicities associated with ICs, which manifest as VLS with hypotension, edema, weight gain (due to fluid retention), serous effusions and hypoxemia in severe cases (8). Indirect immune-mediated mechanisms likely drive IL-2-mediated VLS through activation of T and NK cells, promoting secondary release of inflammatory mediators such as IFN-γ, IL-6, IL-8, and TNF-α, which can in turn injure the endothelium (31, 32). A direct endothelial contribution has also been proposed in some experimental systems, including IL-2 receptor component expression (particularly IL-2 receptor α, IL-2Rα) and barrier perturbation in endothelial cells (33–36). Whether this reflects signaling through a functional high-affinity IL-2 receptor on endothelium *in vivo* remains debated, and immune-cell-mediated mechanisms likely make the dominant contribution to clinical vascular leak (31, 32, 37). In contrast, TNF-α can directly interact with TNFR1/TNFR2 on endothelial cells resulting in disruption of vascular permeability by enzymatic glycocalyx shedding, junctional destabilization and increased actomyosin contractility through complex pathways (38–41). TNF-α can also induce direct cytotoxicity/apoptosis in tissues (42–44). Moreover, indirect TNF-α mechanisms have also been reported to cause endothelial dysfunction (45–47). By contrast, VLS is not a dominant clinical feature of IL-12 ICs, which may in part reflect the absence of the direct or indirect endothelial activation mechanisms associated with IL-2 and TNF-α, although this mechanistic interpretation requires further validation.

Although hypotension, edema and/or hypoxia have been reported for IL-2 ICs targeting GD2 (hu14.18-IL2) (5, 24, 48), EpCAM (huKS-IL2) (6, 26, 27), and fibronectin (L19-IL2) (7, 13, 49–51), these events were mainly mild to moderate. Vascular leak events were most evident with hu14.18-IL2, whereas huKS-IL2 caused mainly low-grade, reversible hypotension (5, 6, 26, 27, 48). Notably, fibroblast activation protein (FAP)-targeting FAP-IL2v showed capillary leak/hypotension despite its abolished IL-2Rα binding, suggesting that IL-2Rα-sparing alone may not eliminate endothelial liability (25). Systemic TNF-α was associated with severe (grade ≥3) VLS events, whereas for L19-TNFα IC, administered via ILP, most of the toxicities were restricted to the limb as expected (10). The outcomes of other TNF-α IC formats remain to be seen.

### 2.2 Infusion-related reactions and cytokine release syndrome

Infusion-related reactions (IRRs) and CRS can both occur shortly after IC administration and may share clinical features such as fever, chills, flushing, rash, hypotension, tachycardia, dyspnea, nausea, and headache. However, the two are not synonymous. IRR is a broad clinical term describing acute adverse signs and symptoms occurring during or soon after infusion and may reflect heterogeneous mechanisms, including hypersensitivity, complement activation, or cytokine-mediated inflammation (52). By contrast, CRS is a distinct systemic inflammatory syndrome driven by supraphysiologic cytokine release and is commonly graded using American Society for Transplantation and Cellular Therapy consensus criteria based primarily on fever, hypotension, and hypoxia (53). Management is generally supportive for low-grade events, whereas higher-grade CRS may require anti-IL-6-directed therapy such as tocilizumab and/or corticosteroids, depending on severity and institutional practice (54). In early-phase IC studies, however, acute events may not always be classifiable cleanly as IRR versus CRS. These toxicities, whilst commonly expected with systemic cytokine therapies, have also been reported with ICs, albeit generally with lower severity.

In clinical studies, IRRs typically seen with IL-2 ICs have varied in intensity. For example, FAP-IL2v reported the highest incidence of IRR, occurring in 65% (22/34) of patients, with 21% (7/34) of patients experiencing grade ≥3 events in the 15/20 mg cohort (25). In hu14.18-IL2 trials, fever, chills/rigors, pruritus and infusion-associated pain have been reported in a high frequency of patients (5). By contrast, mild to moderate IRRs were observed with huKS-IL2 and a DNA-targeting IC NHS-IL2 (6, 26, 27, 55, 56). TNF-α, which carries high inflammatory risk when administered by infusion, often shows a reduced tendency to induce IRR/CRS when administered locally. For instance, IRR/CRS are uncommon with L19-TNFα administered by isolated limb perfusion (10). IRR/CRS toxicities are also not prominent with IL-12 ICs, which may be attributed to their indirect immune-stimulatory mechanism, although current evidence does not establish a definitive causal explanation.

### 2.3 Cytopenias/hematological toxicities

Hematological toxicities, albeit transient, are clinically observed with systemic administration of a range of cytokines (57). Both IL-2 and IL-12 can directly or indirectly induce rapid lymphocyte trafficking into lymphoid tissues/tumors and secondary lymphoid organs by enhancing adhesion molecule (ICAM-1, VCAM-1) or chemokine (CXCL9/10/11) expression (58–60). This redistribution is thought to contribute to acute and reversible peripheral lymphopenia observed clinically. Neutropenia was also reported with certain ICs, particularly when combined with chemotherapies. However, neutropenia is primarily driven by bone marrow suppression from concomitant chemotherapy rather than by ICs (61, 62). Other hematological events associated with ICs include thrombocytopenia and anemia (5, 25, 48). Among other potential mechanisms for cytopenia, IL-2 has been shown to directly suppress circulating erythroid (BFU-E) and granulocytic/macrophage (CFU-C) progenitors, thereby dampening hematopoiesis (63). In addition, IFN-γ, induced by IL-12, TNF-α and IL-2, can induce apoptosis in lymphocytes through Fas/FasL signaling (64). Hypophosphatemia, which is a frequent AE associated with IL-2 ICs, is likely attributed to increased extracellular phosphate uptake by IC-activated lymphocytes which have heightened metabolic demand, a mechanism supported by observations in other T-cell activation settings (65, 66).

IL-2 ICs have frequently been associated with transient but often high-grade lymphopenia. For example, grade 4 events were seen in 64% (9/14) of patients that received hu14.18-IL2 (48). Another noteworthy example was a phase I trial evaluating FAP-IL2v with or without checkpoint inhibitor atezolizumab in which grade 4 lymphopenia was observed in 11/11 patients (67). Similarly, an IL-12 IC was reported to cause grade 2–3 lymphopenia in a first-in-human (FiH) and expansion studies (16, 68). This lymphopenia likely reflects a pharmacodynamic (PD) effect of immune activation rather than direct lymphocyte toxicity. Mild to moderate neutropenia has been reported with IL-2 ICs, when combined with myelosuppressive chemotherapeutics doxorubicin and cyclophosphamide (27, 69). Similarly, grade ≥3 neutropenia in 23% (4/17) of patients was reported with a TNF-α IC when combined with melphalan, a bifunctional alkylating agent with myelosuppressive outcomes (10). Thrombocytopenia and anemia at varying grades have been reported for IL-2 ICs (5, 25, 48). Grade 2–3 hypophosphatemia has been reported following administration of hu14.18-IL2 (5) and FAP-IL2v (25) in 73% (24/33) and 12% (4/34 patients in the 15/20 mg cohort) of patients, respectively.

### 2.4 Immune endocrine toxicities

Thyroid dysfunction has been observed with systemic IL-2 therapy used to treat cancer (70, 71), as hyperstimulation of the immune system can in some cases break down immune tolerance. Whilst not prominent across several single-agent IC cohorts reported to date (8), autoimmunity may still arise when ICs are combined with other modalities. For instance, grade 1–2 thyroiditis was reported in 23% (3/13) of patients treated with NHS-IL2 in combination with radiotherapy in non-small cell lung cancer (56). One of the possible underlying mechanisms is disruption of peripheral immune tolerance, and recruitment of auto-reactive T cells to the thyroid tissue (72–74). Whilst immune endocrine toxicities have not been prominently reported with other IL-2 ICs, this safety risk should not be overlooked in RT and checkpoint inhibitor combinations, as both these approaches are also reported to carry their independent thyroid-related safety risks in the clinic (73, 75).

IC-driven immune activation can also impact the neuroendocrine system, leading to hormonal imbalance and dysregulation in glucose metabolism. It is well established that the cytokines IL-1, TNF-α and IL-6, which are upregulated by IL-2, can activate the hypothalamic–pituitary–adrenal (HPA) axis independently, causing release of cortisol and catecholamines, which in turn lead to hepatic gluconeogenesis and reduced peripheral glucose uptake (76, 77). Conversely, some of these cytokines can also cause peripheral insulin resistance by directly impairing insulin signaling (78–81). Of note, grade 1–3 hyperglycemia (55%, 18/33 grade 1, 15%, 5/33 grade 2, and 9%, 3/33 grade 3) has been reported in patients treated with hu14.18-IL2 (5).

### 2.5 Hepatic injury

Systemic IL-2 and IL-12 therapies have been associated with notable hepatic toxicities, especially with IL-12 where rises in aspartate aminotransferase (AST) and alanine aminotransferase (ALT) were major dose-limiting toxicities (82–87). Hepatic injury associated with both IL-2 and IL-12 likely reflects cytokine-driven, immune-mediated hepatocellular stress rather than direct hepatocyte toxicity. Mechanistically, IL-2 and IL-12 can rapidly induce a proinflammatory milieu (TNF-α, IL-6, IFN-γ), activate Kupffer cells, upregulate adhesion molecules/IFN-γ inducible chemokines (CXCL9/10/11), and recruit CXCR3+ T/NK cells into the liver, which can potentially cause transient necro-inflammatory tissue injury (88–93). Endothelial activation and microcirculatory stress, caused by cytokine surges, may further contribute to hepatocyte injury.

Although the frequency and severity of liver toxicities appear to be lower with IL-2 and IL-12 ICs than historically observed with systemic-cytokine therapy, AST/ALT elevations are still common during treatment (8). For example, significant hepatic injury was reported with FAP-IL2v monotherapy, manifesting in an AST/ALT rise in 24% (8/34) of patients in the 15/20 mg cohort, with dose-limiting grade ≥3 events in 3% (2/61) of patients in all cohorts (25). Other IL-2 ICs such as hu14.18-IL2, L19-IL2 and F16-IL2 (targeting tenascin-C) also presented AST/ALT increases up to grade 3 in monotherapy or combination settings (5, 7, 13, 24, 48, 49, 69, 94). With NHS-IL12, grade ≥3 transaminase elevations were reported as the most prominent AE, but this was reversible (68). It should be noted that FAP-IL2v has since been discontinued on portfolio-prioritization grounds, though its reported safety data remain informative for IC development.

### 2.6 Local inflammatory toxicities

The route of administration of an IC can raise concerns for localized tissue sinks and toxicities depending on the antigen expression profile. For instance, injection-site inflammatory reactions (swelling/erythema) were reported in 67% (4/6) of patients receiving intralesional L19-IL2 (51). This may have been caused by locally-restricted inflammation as the target for L19-IL2, extradomain-B (EDB) fibronectin, is highly expressed in tumors (95). Injection site reactions were either absent or less prominent with intravenous L19-IL2 regimens (13, 50).

Another example of target-mediated toxicity is localized neuropathic pain observed with GD2-targeting IC hu14.18-IL2, wherein 39% (13/33) of patients required opioids for pain management (5). This is particularly relevant in pediatric neuroblastoma, where anti-GD2 therapies are widely used and neuropathic pain is a defining on-target toxicity (96, 97). In this case, binding of GD2-targeting domain to GD2 expressed on the peripheral nerve fibers and induction of local inflammatory responses may potentially contribute to nerve damage (96, 98, 99). Overall, the choice of TAA and route of administration warrant careful consideration during IC development, given the potential for antigen-dependent local toxicities.

## 3 Mitigation strategies

Given the diversity of safety risks described above, multiple non-clinical strategies are being pursued to improve the therapeutic index of ICs. Selected non-clinical strategies that may help de-risk the IC-associated toxicities include cytokine selection, molecular engineering, and approaches to improve targeted delivery to tumor microenvironment (TME) or immune cells (**Figure 1**). These elements are considered in more detail below. Unless otherwise stated, the safety considerations discussed in this section refer to preclinical safety signals, early clinical tolerability observations, or design-based hypotheses rather than established clinical safety advantages. For many emerging IC formats, human safety data remain limited, and proposed tolerability benefits should therefore be regarded as provisional until validated clinically.

**Figure 1:**
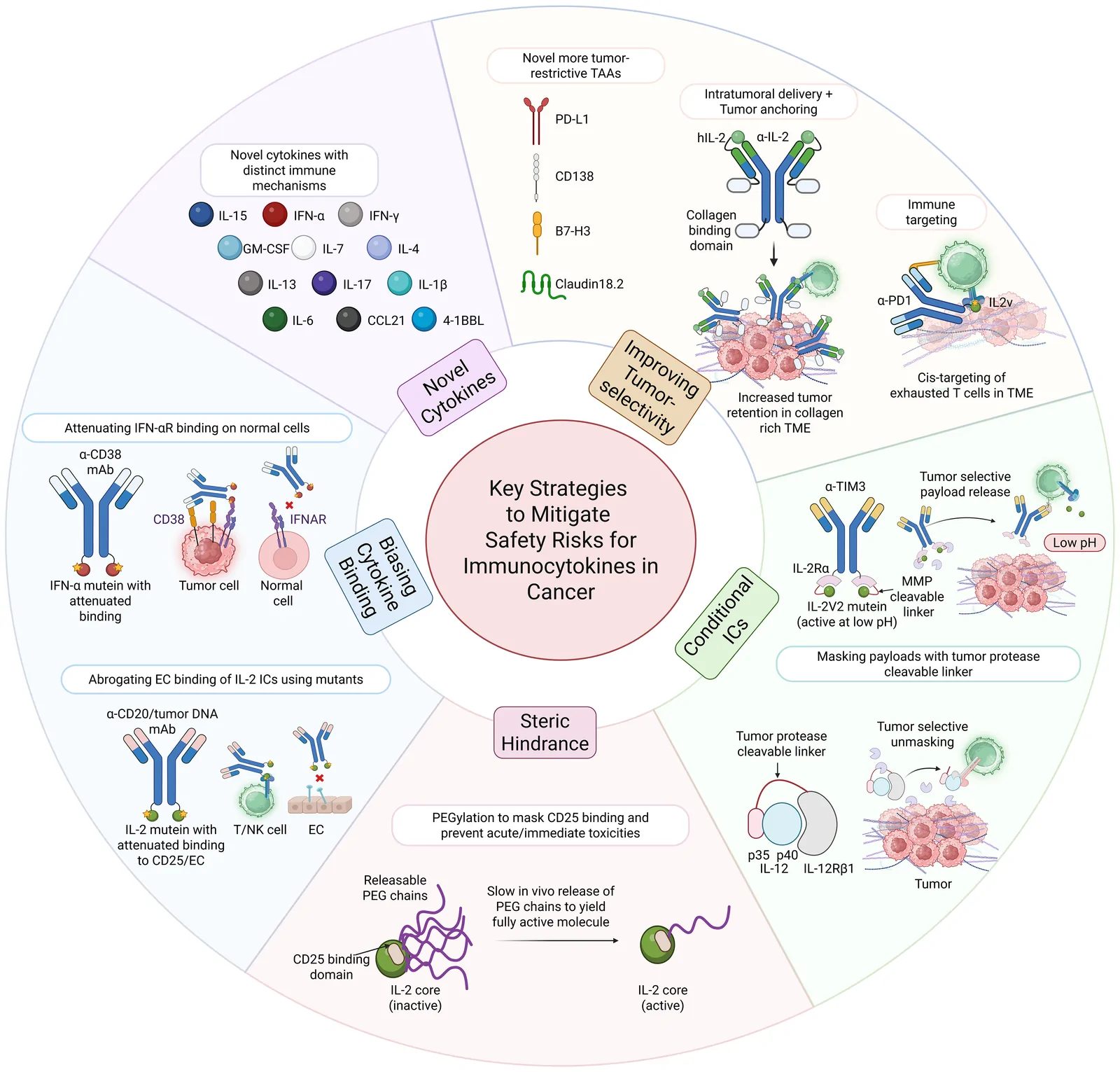
Key strategies to mitigate safety risks associated with immunocytokines in cancer. Schematic overview of five major approaches used to improve the therapeutic index of immunocytokines: novel cytokines, biasing cytokine binding, steric hindrance, conditional ICs, and improving tumor selectivity. These strategies include use of alternative cytokines with distinct immune mechanisms, attenuation of cytokine-receptor binding in normal tissues, transient masking of cytokine activity by PEGylation, tumor-selective activation through low-pH-responsive or protease-cleavable designs, and improved localization through tumor-restricted TAAs, intratumoral delivery with tumor anchoring, or immune-cell targeting. Collectively, these approaches aim to reduce systemic exposure and off-tumor activation whilst preserving antitumor immune activity.

### 3.1 Novel cytokines

A range of alternative cytokines with distinct immunological mechanisms, compared to traditional cytokines described above, are currently being explored (**Table 2**). However, the proposed safety advantages are based on cytokine biology, preclinical models, or limited clinical experience rather than established human data in IC formats. The examples highlighted here are illustrative rather than exhaustive.

**Table 2:** Selected novel cytokines evaluated in immunocytokine preclinical development: proposed safety considerations and reported preclinical data.

Cytokines	TAAs/targets	Safety benefit/evidence	Key references
IL-15	PD-L1 (solid tumors) CD19/CD20 (B-cell malignancies) TA-MUC1 (solid tumors) FAP (solid tumors) GD2 (neuroblastoma) PD-1 (immune-targeting)	• Proposed as a potentially safer IL-2 alternative based on receptor biology and preclinical studies; preferential expansion of CD8+ T cells/NK cells, minimal Treg activation • Emerging focus on masked/prodrug designs to reduce systemic toxicity, checkpoint co-targeting for “cold” tumors, and trifunctional combos • Many preclinical; some (IAP0971, SOT201) in early clinical trials	(136, 137, 139, 204–209)
IFN-α	CD20 (B-cell lymphoma) HLA-DR (lymphoma/myeloma) PD-L1 (solid tumors) EDA/EDB fibronectin variants (solid tumors)	• Targeted IFN-α formats reduce systemic IFN-α-type toxicities (flu-like/cytopenias) relative to untargeted cytokine exposure • Attenuated-binding IFN-α variants (higher receptor dissociation) designed to spare healthy tissues show significantly less off-target toxicity whilst maintaining tumor localization preclinically • ECM (EDA) targeting localized IFN-α to tumor but did not improve control; nonetheless, no added systemic toxicity signal was noted in the preclinical context (safety ≠ efficacy) • Systemic IFN-α therapy was approved by FDA but use limited by DLTs	(9, 120, 210–219)
IFN-γ	EDA fibronectin EDB fibronectin	• EDA/EDB-targeted IFN-γ showed dose-dependent receptor trapping limiting systemic dissemination; reports note no body-weight loss and no acute systemic toxicity signals at active doses in the cited preclinical models	(220, 221)
GM-CSF	GD2 (neuroblastoma/melanoma) HER2 EDB fibronectin	• Safety axis is predominantly myeloid/hematologic (e.g., leukocytosis) rather than acute hemodynamic collapse; targeted formats did not report TNF-/IL-12-like systemic toxicities in preclinical studies summarized	(222–225)
IL-7	EDA fibronectin (solid tumors)	• No serious AEs in clinic with systemic therapy outside the IC setting • Described as a homeostatic cytokine with potentially more manageable acute systemic profile than IL-2/IL-12/TNF • No DLTs observed in cited preclinical mouse models	(206, 226, 227)
IL-4	Experimental tumor-targeted formats (neovasculature combos)	• Type-2 skewing cytokine; potentially lower risk of acute CRS/vascular events than TNF/IL-12 and may avoid IL-2-type capillary leak • Well tolerated in murine models with no toxicities or weight loss	(8, 228, 229)
IL-13	EDA fibronectin	• Similar to IL-4 in toxicity axis (type-2 biology); potentially lower acute hemodynamic risk compared with TNF/IL-12 • No significant toxicity up to 250 µg in mice	(230)
IL-17	EDA fibronectin	• Distinct biology emphasizing autoimmunity/psoriasiform-like inflammation risk • Preclinical targeting promoted angiogenesis without added acute systemic toxicity (no efficacy improvement)	(231)
IL-1β	EDA fibronectin	• Upstream inflammatory mediator: anticipated toxicity is fever/CRS-like local inflammation over vascular • MTD notably lower than IL-6 IC in mice in head-to-head comparison • Targeted delivery is emphasized to mitigate systemic inflammatory toxicity seen with systemic cytokine therapy	(232)
IL-6	EDA fibronectin	• Pleiotropic cytokine biology; acute-phase/metabolic toxicity axis (fever, hepatobiliary effects) expected rather than TNF-like shock or IL-2-type vascular leak • No overt signs of toxicity up to 100 µg dose in preclinical model	(232)
G-CSF	EDA fibronectin	• Predictable, largely manageable toxicities (neutrophilia, bone pain, splenomegaly) in the systemic form and would be expected for ICs based on cytokine biology • IC form is less likely to show acute CRS/vascular profile typical of TNF/IL-12	(229)
CCL21 (chemokine)	Tumor-targeted formats (T cell/DC recruitment; e.g., chemokine/anti-PD-L1 fusions)	• Chemokines are hypothesized to have low propensity for CRS/vascular toxicities • Principal risk is ectopic/local inflammation rather than systemic hemodynamic instability • Available support in immunocytokine format remains limited to preclinical proof-of-concept studies, including *in vitro* targeted delivery and effector-cell recruitment assays	(233)
4-1BBL (TNFSF ligand)	Tumor antigen/ECM-anchored co-stimulation (FAP in trifunctional formats)	• Systemic 4-1BB agonism is associated with hepatotoxicity • Targeted/anchored 4-1BBL formats are being explored with the aim of reducing liver toxicity; distinct from IL-2/TNF/IL-12-type systemic risks. • No overt toxicity observed in syngenic model.	(234, 235)

• Examples shown are representative of approaches discussed in the text and are not intended to provide an exhaustive inventory of all reported immunocytokine formats.

• Safety considerations summarized in this table are based on preclinical studies and/or mechanistic rationale and should not be interpreted as established clinical safety advantages in humans.

AE, adverse event; ALT, alanine aminotransferase; AST, aspartate aminotransferase; 4-1BBL, 4-1BB ligand; CCL21, C-C motif chemokine ligand 21; CD19, cluster of differentiation 19; CD20, cluster of differentiation 20; CRS, cytokine release syndrome; DC, dendritic cell; DLT, dose-limiting toxicity; ECM, extracellular matrix; EDA, extra-domain A; EDB, extra-domain B; FAP, fibroblast activation protein; Fc, fragment crystallizable region; FDA, Food and Drug Administration; G-CSF, granulocyte colony-stimulating factor; GD2, disialoganglioside GD2; GM-CSF, granulocyte-macrophage colony-stimulating factor; HER2, human epidermal growth factor receptor 2; HLA-DR, human leukocyte antigen-DR; IC, immunocytokine; IFNAR, interferon alpha receptor; IL-2R, interleukin-2 receptor; NHP, non-human primate; NK, natural killer; PD-1, programmed cell death protein 1; PD-L1, programmed death-ligand 1; TA-MUC1, tumor-associated mucin 1; TAA, tumor-associated antigen; TME, tumor microenvironment; TNFSF, TNF superfamily.

Type I and type II interferons (IFN-α and IFN-γ), IL-15, IL-7, IL-4, IL-13, IL-17, IL-21, GM-CSF, G-CSF, chemokine CCL21, and 4-1BBL may avoid some of the major toxicities (e.g. VLS, hematological AEs) seen with IL-2 whilst preserving useful immune stimulation, although clinical validation remains limited (100–108). These alternative cytokines are not expected to reproduce TNF-like endothelial injury and IL-12-like systemic IFN-γ cascades to the same extent, which remains to be demonstrated clinically. Another goal of the alternative cytokine selection is to bias immune activation towards priming and durability. For example, IL-7 and GM-CSF enhance long-term homeostasis and antigen presentation, which in theory could translate into a different and potentially more manageable acute toxicity profile whilst providing comparable immune stimulation (101, 109–113). Moreover, these cytokines may introduce additional safety considerations owing to their distinct biology.

### 3.2 Molecular design

#### 3.2.1 Modified cytokine binding

Multiple groups have engineered ICs with IL-2 antibody complexes to abrogate binding to IL-2Rα and thereby reduce signaling through the high-affinity IL-2 receptor (34, 114–119). One such IC, NARA1leukin, demonstrated no severe IL-2-mediated toxicities in mice (118). Most recently, Leonard and Tomala et al. tethered IL-2 with IL-2Rα-competitive anti-IL-2 biasing antibody to form an intramolecularly-assembled IC, which was devoid of major IL-2 associated AEs such as acute pulmonary and liver toxicities (AST/ALT changes) in preclinical models (119). Reducing the binding affinity to cytokine receptors expressed in normal tissues can also potentially widen the therapeutic index. For instance, an IFN-α IC with attenuated IFN-α-receptor (IFNAR) binding affinity was found to be 6000-fold less toxic to bone marrow cells and had significantly reduced IFN-α-biomarker activity in healthy non-human primates (NHPs) (120).

ICs incorporating IL-2 muteins that disrupt binding to either endothelial cells or IL-2Rα have shown encouraging preclinical results and some early clinical promise for improved tolerability. IL-2 muteins that preferentially bind intermediate affinity IL-2 receptor (IL-2Rβγ) showed reduced Treg expansion, absence of VLS, and an improved overall safety profile in mouse models across multiple target settings such as carcinoembryonic antigen (CEA), programmed cell death protein 1 (PD-1), cluster of differentiation 20 (CD20), and an undisclosed tumor-target (18, 121–123). NHS-IL2, a DNA-targeting IC carrying an IL-2 mutein with abrogated binding to endothelial cells (but not IL-2Rα), exhibited a superior safety profile compared to unmodified IL-2 IC in phase I trials with no severe capillary leak syndrome/hypotension, or life-threatening events (55, 124–126).

#### 3.2.2 Tethering and steric hindrance

Besides tumor-targeting ICs, it is worth noting another interesting approach to limit systemic toxicity of IL-2, wherein IL-2 activity is attenuated *in vivo* through conjugation to multi-arm polyethylene glycol (PEG), designed to release active IL-2 moieties progressively. NKTR-214 (bempegaldesleukin), a PEGylated IL-2 prodrug designed to attenuate rapid immune activation/toxicities, showed a meaningfully improved safety profile in patients compared to systemic IL-2, with mostly low-grade AEs and no evidence of VLS and hypotension in trials evaluating monotherapy and in combination with a checkpoint inhibitor (127–133). The development of NKTR-214 was discontinued following failure to demonstrate efficacy in phase III trials (130).

#### 3.2.3 Conditional ICs

An important approach to emerge in the last 5 years is conditional ICs, wherein the cytokine domain in the IC is most commonly masked using a tumor protease-cleavable linker to expose or activate full cytokine functionality selectively in the TME. Recently, Song et al. developed a universal Fab-Fc masked cytokine prodrug platform, which uses steric hindrance of Fab/Fc antibody domains to mask cytokine activity in the absence of tumor proteases with improved tolerability (134). Several conditional ICs have shown encouraging safety profiles that are summarized in **Table 3**. However, for the majority of these molecules, available evidence is preclinical, and in some cases, is available only in conference abstracts or early translational reports.

**Table 3:** Selected conditional immunocytokines and their reported preclinical safety findings.

Molecule name	Molecular design	Safety evidence	References
ProIL2	Heterodimeric Fc-fusion prodrug: CD8+-biased IL-2 superkine (SumIL2 mutein with F42A) masked by full IL-2Rβ extracellular domain via MMP-cleavable linker	Equivalent/superior antitumor efficacy vs. unmasked; no significant body-weight loss, suppressed serum inflammatory cytokines (IFN-γ, MCP-1), significantly lower liver enzymes (AST/ALT), reduced pulmonary edema, minimal off-target organ infiltration; no hematologic or organ toxicities were reported in preclinical mouse model	(236)
PD1-IL2Ra-IL2 (REGN10597)	Anti-PD-1 IgG4P (Fc-silenced) with wild-type IL-2 fused in cis to IL-2Rα extracellular domain mask via flexible (GGGGS) linker	No body-weight loss and pulmonary edema compared to unmasked molecule in syngeneic model; normal serum cytokines, no liver/kidney toxicities or treatment-related mortality; systemic IL-2 activity attenuated on non-PD-1+ cells	(156)
M-L_6_-IL12	IL-12 fused to IL-12Rβ1 (first two fibronectin type-III domains) mask via MMP-cleavable linker	No body-weight loss at 3-fold molar dose of systemic IL-12; plasma cytokines (IFN-γ, IL-6, TNF-α, CCL2) reduced to near PBS levels; liver (ALT/AST) and pancreas enzymes significantly decreased; no leukopenia/lymphopenia in healthy mice	(237)
Protease-cleavable masked IL-12 (CytomX, Moderna)	mRNA encoded masked IL-12: lead peptide mask attached via protease-cleavable linkers	No body-weight loss, normal liver weights, no mortality up to 0.1 mg/kg (30-fold higher than lethal dose of unmasked IL-12 mRNA); plasma IFN-γ remained <5,000 pg/mL; durable complete responses without overt toxicity reported	(238)*
MDNA113 (BiSKIT)	Bifunctional anti-PD-1 antibody fused to IL-13 Superkine that masks IL-2; protease-sensitive linker cleaved by TME MMPs to release active anti-PD1-IL-2 superkine	Strongly attenuated peripheral IL-2R signaling in the absence of cleavage; better tolerated than unmasked; no weight loss or overt toxicities reported in murine tumor models	(239)*
Pro-IL-12	Shielded/tumor-conditional IL-12 masked with selective extracellular receptor-binding domains of the IL-12 receptor via MMP-cleavable linkers	Strong antitumor efficacy with limited toxicity vs. recombinant IL-12; improved preclinical safety profile whilst enhancing checkpoint blockade/combinations; no profound body-weight loss or organ damage at efficacious doses	(240)
IL-2/IL2Rα fusion protein (IL-2FP)	Mouse IL-2 fused to IL-2Rα via an MMP2/9-specific protease-cleavable linker	Attenuated systemic activity pre-cleavage; reduced peripheral immune activation and cytokine storm vs. systemic IL-2; improved tolerability whilst preserving antitumor efficacy in mouse models (no detailed body-weight, organ, or cytokine data specified)	(241)
TIM3-ProIL2V2	Anti-TIM3-Fc fused to pH-selective IL-2V2 (higher β affinity at acidic pH) via MMP-cleavable IL-2Rα mask	No significant weight loss/side effects in mice in contrast to same molecule without conditionality (MMP cleavage); remarkably less IFN-γ release *in vivo* compared to unconditional molecule	(167)
cLAG3-IL2	LAG3 targeting IL-2 with conditionality (details undisclosed)	No signs of typical IL-2-mediated toxicities reported in cited preclinical model	(168)*
MiTE (Myeloid-targeted Immunocytokines and NK/T-cell Enhancers)	Anti-TREM2 (antagonistic) antibody fused to masked IL-2 carrying linker cleavable by MMP14 proteases in TME	No systemic inflammation, hepatotoxicity, or weight loss in mouse models; no major cytokine spikes in IFN-γ, IL-2, and IL-6; by contrast, unconditional IC led to severe systemic inflammation and hepatotoxicity	(171)

• Examples shown are representative of approaches discussed in the text and are not intended to provide an exhaustive inventory of all reported immunocytokine formats.

• Safety considerations summarized in this table are based on preclinical studies and/or mechanistic rationale and should not be interpreted as established clinical safety advantages in humans.

* Non-peer-reviewed conference abstracts.

ALT, alanine aminotransferase; AST, aspartate aminotransferase; CCL2, C-C chemokine ligand 2; IC, immunocytokine; IgG4P, immunoglobulin G4 proline-substituted (Fc-silenced); IL-12Rβ1, interleukin-12 receptor beta 1; IL-2R, interleukin-2 receptor; IL-2Rβ, interleukin-2 receptor beta subunit (CD122); LAG3, lymphocyte-activation gene 3; MCP-1, monocyte chemoattractant protein-1; MiTE, myeloid-targeted immunocytokines and NK/T-cell enhancers; MMP, matrix metalloproteinase; MMP14, matrix metalloproteinase 14; NK, natural killer; PBS, phosphate-buffered saline; PD-1, programmed cell death protein 1; TIM3, T-cell immunoglobulin and mucin-domain containing-3; TME, tumor microenvironment; TREM2, triggering receptor expressed on myeloid cells 2.

### 3.3 Improving tumor selectivity and immune-targeting ICs

TAAs that have highly restricted expression in tumors (and minimal normal tissue levels) are currently being evaluated preclinically as alternatives to classical targets like fibronectin EDB, tenascin-C, necrotic DNA, and stromal/extracellular matrix components (8). Key examples include programmed death-ligand 1 (PD-L1) for multiple tumors (135–140), syndecan-1 (CD138) for multiple myeloma [ (141), and non-peer-reviewed abstract, (142)], B7 homolog 3 protein (B7-H3) for glioblastoma [(143), and non-peer-reviewed abstract, (144)], and claudin 18.2 for gastric/pancreatic cancer (145). These TAAs could potentially enable more precise tumor localization of the cytokine moiety and minimize systemic, off-target toxicities.

Intratumoral delivery of immune-based therapies has also been demonstrated as a viable clinical strategy for some cancers to reduce systemic cytokine-related AEs (146–149), although wider distribution occurs due to the vascularized nature of the tumor environment (147, 150, 151). Recent studies have shown that anchoring ICs to collagen via linkage to collagen-binding domain improves retention in collagen-rich TME, with subsequent reduction in systemic exposure and off-target toxicities in the context of intratumoral delivery (152, 153).

In recent years, cis-targeting ICs have emerged whereby the cytokine is delivered directly to TME-specific immune cells instead of tumor antigens or extracellular matrix, offering an effective way to minimize off-target effects and enhance efficacy (154). PD-1-guided IC, designed to reinvigorate exhausted CD8+ T cells, have emerged as a prominent example of this approach with growing preclinical evidence suggesting a favorable safety profile (154–157), with additional support from a conference abstract (non-peer-reviewed) (158) and some early clinical data suggesting good tolerability [(121, 159), and non-peer-reviewed abstracts (160, 161)]. However, these observations remain preliminary and are not yet sufficient to establish a class-wide clinical safety advantage. Furthermore, multiple CD8-directed ICs are also in preclinical development [(154, 162–165), and non-peer-reviewed abstract, (166)]. AB248, the most advanced CD8-guided IL-2 IC, has demonstrated greater than 500-fold selectivity for CD8+ T cells with improved tolerability in preclinical mouse models and selective CD8+ expansion in NHPs (162, 163). Whether these findings translate into a clinically meaningful safety advantage remains under evaluation in early-phase trials [non-peer-reviewed abstract, (164)]. Other emerging targets based on cis-targeting approaches are T-cell immunoglobulin and mucin-domain containing-3 (TIM3) (167), lymphocyte-activation gene 3 (LAG3) [non-peer-reviewed abstract, (168)], T-cell receptor variable β chains (169), and leukocyte common antigen (CD45) (170). Recently, a trans-targeting approach named MiTE (myeloid-targeted immunocytokines and NK/T-cell enhancers) was introduced by von Locquenghien et al. (171). This approach comprises an anti-triggering receptor expressed on myeloid cells 2 (TREM2) antibody linked to masked IL-2 capable of stimulating T and NK cells in trans-fashion after unmasking by matrix metalloproteinase 14 in the TME (171). MiTE has shown an encouraging safety profile preclinically (**Table 3**), but clinical safety data are not yet available.

### 3.4 Other strategies

Changing IC delivery route to intratumoral, as discussed above (152, 153, 170), can help limit off-target effects if paired with the right tumor-anchoring approaches in some settings, although this approach is constrained by disease type and accessibility to tumors. Fc engineering or use of Fc-free design (e.g. use of single-chain variable fragment, scFv) is also a common strategy across the spectrum of biologics to lower FcγR binding and complement activation, and thereby minimize cytotoxicity on FcγR expressing normal cells (9, 172, 173).

## 4 Immunogenicity

Immunogenicity is an important challenge for ICs, which carry a higher theoretical risk than many other biologics owing to their complex multidomain structures. Modified antibody scaffolds (e.g. scFv, IgG), linker-junction architecture, and engineered cytokine mutein domains can create immunogenic neoepitopes, leading to the generation of anti-drug antibodies (ADAs). Moreover, a functional cytokine moiety can activate antigen-presenting cells as well as B and T cells, thereby enhancing immunogenicity. Clinically, immunogenicity may alter PK, reduce drug exposure and efficacy, and in some cases contribute to Fc-mediated immune adverse reactions such as hypersensitivity, anaphylaxis, and immune-complex formation (174).

Clinical experience indicates that some ICs can elicit frequent ADA responses, sometimes with clear PK consequences. The highest incidence of immunogenicity was reported with cluster of differentiation 38 (CD38)-IFNα, which induced ADAs in 100% (39/39) of patients in a phase I/II trial, with neutralizing ADAs (nADAs) in 82% (14/17) and 91% (10/11) of evaluable patients in phases I and II, respectively (175). This ADA response was associated with reduced drug exposure and may have contributed to the limited efficacy of CD38-IFNα (175). The EpCAM-targeting IC huKS-IL2 also showed frequent transient ADA formation in phase I and II studies, including anti-idiotype antibodies and antibodies targeting Fc-IL-2 regions (27). By contrast, a phase I study of NHS-IL2 reported transient ADA responses in 69% (33/48) of patients, but without apparent effects on PK or PD (55). Administration of hu14.18-IL2 led to anti-idiotype antibodies in 52% (32/61) of patients and antibodies against the Fc-IL2 region in all patients, but was not associated with increases in toxicity or allergic phenomena (176).

Perhaps the most direct exposure-immunogenicity relationship was reported for CEA-IL2v, which comprises an IL-2 variant with abolished binding to IL-2Rα. In a phase I trial, CEA-IL2v elicited ADAs in 84% (43/51) of evaluable patients with advanced/metastatic solid tumors, and these were associated with decreased drug exposure in most patients (177). Although no clear impact on safety was observed, this may have contributed to the limited clinical activity of the agent. Notably, pretreatment with obinutuzumab before CEA-IL2v administration prevented ADA formation in all 15 evaluated patients, compared with 67% (4/6) of patients showing ADA formation without obinutuzumab (177).

In contrast, several ICs have shown low or clinically limited immunogenicity despite repeated dosing. Fully human L19-IL2 showed no detectable human anti-fusion protein antibodies in phase I and II studies (7, 49). Similarly, no F16-IL2-specific human anti-fusion protein antibodies were detected in serial samples from all 19 patients after repeated exposure, aside from one transient low-level signal by surface plasmon resonance (69). For FAP-IL2v, ADAs were detected across dose levels in a phase I trial, but unlike CEA-IL2v, they showed no notable effect on drug exposure (25).

Taken together, these data suggest that immunogenicity of ICs is not a uniform class effect, but a design-dependent liability shaped by molecular format and engineering. Fully human ICs comprising small-format, Fc-free structures such as scFvs (e.g. L19-IL2, F16-IL2) or diabodies appear to show lower immunogenicity than ICs with humanized IgG-based constructs (e.g. huKS-IL2, CD38-IFNα), likely because of lower foreign epitope burden and reduced antigen presentation. These observations also indicate that humanization alone may be insufficient. Beyond molecular format, preclinical evidence also suggests that route of administration may influence functional immunogenicity. Intratumoral hu14.18-IL2 reduced ADA-mediated neutralization relative to intravenous dosing in a mouse melanoma model whilst maintaining antitumor activity (178). However, translatability to the clinic remains to be established. Furthermore, due to discrepancies in ADA assay format and reporting thresholds across studies, different dosing routes and schedules, and other clinical variables, cross-trial comparisons remain imperfect.

## 5 Translational safety

A translational safety strategy for ICs should integrate species relevance, tissue cross-reactivity, human *in vitro* hazard assessment, and model-informed FiH dose selection, whilst recognizing the limitations of each component.

### 5.1 Selection of toxicology species

A central consideration in IC development is the assessment of species cross-reactivity and selection of a pharmacologically relevant species for *in vivo* toxicology. For ICs, the pharmacology of both components must be established: the antibody moiety should bind the target antigen, and the cytokine domain should activate the cognate cytokine receptor complex with sufficiently similar potency and signaling characteristics. This dual requirement frequently complicates species selection, as cytokine-receptor interactions are often species restricted.

For some human cytokines, murine models are poorly informative, particularly with engineered cytokines where modified engagement is not sufficiently recapitulated (179, 180). NHPs are commonly used as the non-rodent toxicology species because of the relative conservation of cytokine receptor biology and FcRn-mediated PK. Cross-reactivity of IL-2 receptor biology has been demonstrated in NHPs (181). For example, FAP-IL2v retained high-affinity binding to IL-2Rβγ across human and NHP (19). Likewise, NHS-IL2LT, a low-toxicity IL-2 mutein IC, was evaluated in a NHP GLP toxicology study without the severe toxicity historically associated with parental IL-2-based constructs (125). NHPs have also been used for IL-12 IC toxicity studies, supported by comparable IL-12R expression and functional responses (182, 183). In one study, NHS-IL12 induced less IFN-γ in NHPs than recombinant human IL-12, consistent with an attenuated systemic inflammatory profile (184). Together, these examples show that species selection should, wherever possible, be supported by empirical cross-reactivity and functional activity data for both the antibody target and cytokine receptor before GLP studies are initiated, consistent with ICH S6(R1) (185).

When cross-reactivity of one or both IC components is absent in conventional toxicology species, a surrogate-molecule or humanized/transgenic rodent models may provide supporting data. For example, a bivalent anti-NHP CEA surrogate was used for CEA-IL2v because the clinical construct did not cross-react with cynomolgus CEA (18). Although pragmatic, such approaches introduce translational uncertainty because the surrogate may differ from the clinical molecule in binding, immunobiology, and PK. In some cases, a multimodal strategy may therefore be more appropriate, combining human *in vitro* systems for pharmacology and cytokine-release liability, surrogate syngeneic models for tumor-localized biology, and limited *in vivo* studies in the closest cross-reactive species for PK/PD, tolerability, and pathology.

Despite their widespread use, NHP studies have important translational limitations. Healthy NHPs are tumor-free, so tumor-associated antigen expression may be absent or minimal, and cytokine receptor biology may differ quantitatively from that of oncology patients. In addition, healthy NHPs typically have a more quiescent immune system, which may cause studies to underpredict immune-mediated toxicities (186). This limitation may be amplified when surrogates are required. For example, an anti-NHP CEA surrogate for CEA-IL2v did not produce vascular leak or hepatotoxicity in toxicology studies, whereas this molecule was associated with dose-limiting capillary leak syndrome and elevations in liver enzymes in the clinic (187).

### 5.2 Tissue cross-reactivity studies

After species selection, an important next step is to assess whether the targeting arm shows unintended binding in normal human tissues. As some TAAs are also expressed at low levels outside tumors, this can result in on-target, off-tumor toxicities (188, 189). Tissue cross-reactivity (TCR) studies are employed to identify potential normal-tissue binding liabilities. Typically, immunohistochemistry is performed on fresh-frozen normal human tissues from at least three donors using a broad tissue panel (190). For ICs, TCR interpretation is more complex than for conventional antibodies because receptors for the employed cytokines may be present on circulating and tissue-resident immune cells, creating background staining when the intact fusion protein is used. TCR is therefore often more informative when performed with the antibody moiety alone, but even then, a positive result does not establish clinical risk by itself, and a negative profile does not exclude cytokine-mediated toxicity. More recently, membrane protein arrays (MPAs) have been employed to assess off-target binding liabilities of antibody-based therapeutics (191). However, as noted in ICH S6(R1), binding alone does not establish *in vivo* biological activity. TCR and MPA findings therefore need to be interpreted in the context of pharmacology, biodistribution, and toxicology (185), and are best complemented by *in vitro* assays with human cells.

### 5.3 Cytokine release assays

CRS is among the most important acute safety risks for ICs. Cytokine release assays (CRAs) are widely used for early hazard identification with antibody-based therapeutics, where appropriate. Formats include peripheral blood mononuclear cell- and whole-blood-based assays performed in soluble-phase or solid-phase configurations (192–194). Whilst conventional CRAs can assess unintended cytokine release with immunostimulatory antibodies, the addition of the cytokine component to such assay systems when testing an IC can complicate interpretation. Therefore, it is more informative to assess the antibody component alone in a CRA. Assessment of cytokine-mediated toxicity risk of an IC should be conducted in models that incorporate immune cells together with endothelium, stromal cells and other tissue-relevant components. For example, Zimmermann et al. described an endothelial-peripheral blood mononuclear cells model that reproduced key features of IL-2-induced capillary leak syndrome together with inflammatory cytokine release (37). More advanced systems, including 3D multicellular co-cultures, organoids, and organ-on-chip platforms, may further improve physiological relevance by incorporating vascular, stromal, tissue-specific, and immune components (195, 196) and provide readouts beyond soluble cytokines, such as barrier integrity, endothelial activation, and immune-cell activation states. Whether these systems will prove more predictive for IC-associated CRS or other toxicities than traditional assays remains to be established.

### 5.4 First-in-human dose selection

Selection of the FiH dose is a critical step in IC development. For potent immune-stimulatory biologics, a minimum anticipated biological effect level (MABEL)-based approach is recommended as the primary framework for FiH dose selection, complementing rather than relying on a no observed adverse effect level (NOAEL)-based approach alone (197). This is because cytokines can drive steep or non-linear dose–response relationships, potentially leading to AEs such as cytokine storm and vascular leak at exposures not predicted by standard animal toxicology studies. FiH dose selection for ICs should therefore be grounded in human-relevant pharmacology and integrated with available animal data rather than derived solely from conventional toxicology margins (198).

MABEL determination generally integrates the most sensitive human-relevant PK/PD inputs, including *in vitro* or ex vivo pharmacology, CRA data, receptor occupancy modeling, and exposure prediction accounting for target-mediated drug disposition and tumor sink effects (199, 200). Because this framework may yield a conservative starting dose (200), model-informed approaches such as quantitative systems pharmacology (QSP) are becoming increasingly relevant for FiH dose selection for complex immunotherapies, particularly when relevant animal models are limited (201, 202).

## 6 Conclusions and future perspectives

Clinical and preclinical experience with ICs now suggests that the central challenge is no longer simply whether cytokines can be retargeted to tumors, but whether cytokine activity can be controlled with sufficient spatial, cellular, and temporal precision to improve the balance between activity and toxicity. First-generation ICs established proof of concept by showing that antibody-guided delivery of cytokines such as IL-2, IL-12, and TNF-α can widen the therapeutic window relative to systemic cytokine therapy. In practical terms, this has often meant that ICs can be administered at higher clinically effective exposures than systemic cytokines, thereby enabling stronger pharmacologic activity and, in some settings, improved antitumor efficacy. An additional advantage is an improved pharmacokinetic profile, primarily a prolonged half-life relative to free cytokines, which can support lower or less frequent dosing than would be feasible with systemic cytokine therapy whilst maintaining biologically meaningful exposure. Nonetheless, clinically relevant dose-limiting toxicities associated with systemic cytokine therapy are not fully abrogated by ICs.

The field is therefore moving beyond simple tumor-targeting toward increasingly sophisticated control of cytokine biology. A second-generation of ICs has emerged in which attenuated or receptor-biased cytokine muteins, improved target selection, and format engineering are used to reduce signaling in liability-associated normal tissues whilst preserving useful immune activation at tumor sites. A third-generation is also taking shape through tumor-conditional masking, protease-activatable designs, cis-targeting to defined immune populations such as PD-1+ or CD8+ T cells, and potentially dual-cytokine domain architectures that combine complementary immune functions within a single construct. The importance of these approaches lies not only in their potential to reduce systemic toxicity, but also in their ability to better restrict where, when, and on which cells cytokine activity is delivered.

This shift is important because the future clinical role of ICs will likely depend less on their use as stand-alone delivery vehicles for cytokine monotherapy and more on whether they can overcome both toxicity and efficacy barriers sufficiently to support rational combinations. In principle, these formats may help improve effector-cell persistence and function within the suppressive TME, but the extent to which this can be achieved clinically remains uncertain. This is particularly relevant for combinations with immunotherapy amplifiers such as checkpoint inhibitors, radiotherapy, T-cell engagers, and potentially cellular therapies such as CAR T cells, where overlapping or additive primary inflammatory, endothelial, hepatic, or hematologic toxicities may become dose-limiting.

At present, many of the most innovative directions in the field, including conditional masking, cis-targeting, and dual-cytokine formats to further attenuate IC toxicities, are mainly supported by preclinical evidence. Their proposed advantages therefore require clinical confirmation. Progress will depend not only on molecular engineering, but also on rigorous translational derisking, including appropriate species selection, human-relevant hazard assessment, safe FiH dose selection, and management of immunogenicity. If these hurdles can be addressed, ICs may become more tractable components of next-generation combination immuno-oncology regimens.
